# The potential of mitochondrial transfer as the modifying therapy for osteoarthritis

**DOI:** 10.3389/fcell.2025.1643141

**Published:** 2025-08-26

**Authors:** Yuanpei He, Xinge Wang, Boya Xu, Shichao Chen, Hongcai Li, Bei Chang, Chao Hu, Xiaorong Lan, Shiting Li, Guangwen Li

**Affiliations:** ^1^ The Affiliated Stomatological Hospital, Southwest Medical University, Luzhou Key Laboratory of Oral and Maxillofacial Reconstruction and Regeneration, Luzhou, China; ^2^ Department of Stomatology, Norman Bethune International Peace Hospital, Shijiazhuang, China; ^3^ School of Stomatology, The Fourth Military Medical University, Xian, China; ^4^ Department of Stomatology, The 941 Hospital of the Joint Service Support Force of the People’s Liberation Army of China, Xining, China; ^5^ Department of Stomatology, Shuguang Hospital Affiliated to Shanghai University of Traditional Chinese Medicine, Shanghai, China; ^6^ The PLA Rocket Force Characteristic Medical Center, Beijing, China; ^7^ Department of General Dentistry, The Integrated Traditional Chinese and Western Medicine Hospital, Wenzhou Medical University, WenZhou, China

**Keywords:** mitochondrial transfer, osteoarthritis, disease-modifying, therapymitochondrial dysfunction, artificial mitochondrial transfer

## Abstract

Mitochondrial transfer is defined the process through which specific cell types release their mitochondria and subsequently transfer them to unrelated cell types in response to various physiological or pathological stimuli. This process enhances cellular function and alters disease states. Recent research has begun to explore the potential of intercellular mitochondrial transfer as a therapeutic strategy for human diseases. Mitochondrial dysfunction represents a significant pathological alteration in osteoarthritis, and studies indicate that mitochondrial transfer may serve as an effective modulatory treatment approach for osteoarthritis. Mitochondrial transfer, as an innovative subcellular therapeutic technique, presents the advantages of diverse acquisition methods and multiple transmission pathways. This paper aims to summarize the current understanding of the mechanisms of mitochondrial transfer in relation to osteoarthritis, emphasizing the existing research on mitochondrial transfer in osteoarthritis and its potential as a disease-modifying therapy.

## 1 Introduction

Within eukaryotic cells, mitochondria are considered some of the earliest internal membrane systems. They create adenosine triphosphate (ATP) through the mechanism of oxidative phosphorylation (OXPHOS), thus supplying the essential energy for cell functions and serving as a key driving force for numerous cellular processes. This is why they are commonly referred to as the “powerhouses of the cell” (Lane and Martin, 2010). Mitochondria were initially recognized for their critical role in OXPHOS, but as a diverse family of organelles, they exhibit a multitude of additional functions—up to a dozen—beyond OXPHOS, all of which are essential for cellular viability. To systematically delineate these various functions, researchers have categorized them into five dimensions: molecular level, biological characteristics, bioactivity, functions, and biological behaviors, Furthermore, they have established three levels of histological organization, which include cell types and subtypes, and mitochondrial characterization (Monzel et al., 2023). Mitochondrial functions encompass a range of molecular activities, including amino acid metabolism (Ryu et al., 2024)^.^, ion uptake (Naón et al., 2023), and molecular damage (Verkerke et al., 2024); biological characteristics related to protein input (Tracy et al., 2022), lipid synthesis (Wedan et al., 2024), molecular modifications (Zhang F. et al., 2023; Miriam et al., 2021), and the maintenance and expression of mitochondrial DNA (mtDNA) sequences (Saurer et al., 2023; Rahul et al., 2023); bioactivity categories involving transport (Zhu et al., 2024) and protein complexes/supercomplexes (Milenkovic et al., 2023); functional aspects including OXPHOS (Manford et al., 2021), permeability transition (Zhou et al., 2019)^.^, and the biosynthesis of iron-sulfur (Fe/S) clusters and hormones (Antoine et al., 2017; Olga et al., 2025); and biological behaviors such as fusion/fission dynamics (Di Bona et al., 2024), mitotic nuclear signaling (Zierhut et al., 2019), and intercellular transfer (Baldwin et al., 2024). These categories illustrate a hierarchical progression of mitochondrial functional behaviors, wherein higher-level biological functions exert regulatory feedback on lower-level biological functions.

Mitochondrial transfer is defined as the targeted release of functional mitochondria from donor cells and their subsequent uptake by recipient cells, modulating critical cellular activities (Borcherding and Brestoff, 2023; Frisbie et al., 2024). Research has demonstrated that intercellular mitochondrial transfer can occur through multiple mechanisms in different tissues, serving several critical functions, including supporting the metabolism of recipient cells (McCully et al., 2009; Caicedo et al., 2015; Kim et al., 2018), regulating cell quality (Hutto et al., 2023; Huang et al., 2023), promoting wound healing (Boudreau et al., 2014), modulating the immune system (Tanmoy et al., 2021), and maintaining metabolic homeostasis (Brestoff et al., 2021; Crewe et al., 2021). Mitochondrial transfer provides novel insights for disease treatment: if extracellular mitochondrial transfer into cells is indeed feasible, it raises the question of whether we can artificially introduce free mitochondria via specific pathways to modulate physiological processes or to influence the occurrence and progression of diseases. Consequently, research pertaining to mitochondrial transplantation has gradually begun to emerge (Lin et al., 2024; Wu et al., 2024). Mitochondrial transplantation involves the replacement or compensation of damaged mitochondria within cells with healthy mitochondria, thereby impacting cellular metabolism, signal transduction, and promoting cell survival. Numerous studies have investigated mitochondrial transplantation utilizing various carriers (Cai W. et al., 2024; Long et al., 2024; Sun et al., 2022), establishing a robust research foundation for the treatment of diverse diseases, such as wound healing (Yao et al., 2024), pulmonary diseases (Ding et al., 2024), and cancer (Marabitti et al., 2024), while providing promising therapeutic directions.

Mitochondrial transfer is regulated by specialized intercellular mechanisms that exhibit precise tissue and cell type selectivity, directly modulating essential cellular functions (Table1) (Baldwin et al., 2024; Levoux et al., 2021; Gao et al., 2019; Feng et al., 2023; Wang et al., 2020; Fahey et al., 2022; Rosina et al., 2022; Brestoff et al., 2021; Kawano et al., 2023; Jacoby et al., 2022; Mistry et al., 2019; Ritsuko et al., 2024; Yuan et al., 2021; Morrison et al., 2017; Nicolás-Ávila et al., 2020; Ikeda et al., 2021; Zhou et al., 2024; van der Vlist et al., 2022; Eo et al., 2024; Peruzzotti-Jametti et al., 2021; Jia et al., 2023; Norat et al., 2023; Weinhäuser et al., 2023; Kidwell et al., 2023; Zhang H. et al., 2023; Lazar and Goldfinger, 2021; Xu et al., 2021a). At the cellular level, mitochondrial transfer can enhance the energy metabolism of recipient cells, maintain homeostasis, and activate cellular functions (Cai J. et al., 2024). Notably, cancer cells are known to actively acquire mitochondria from immune cells such as T-cells and macrophages. The acquisition of mitochondria by cancer cells can weaken anti-tumor immunity, support their metabolic demands, and promote proliferation (Zhang H. et al., 2023). At the tissue level, mitochondrial transfer contributes to the stability of various organ systems and initiates coordinated defense and protective mechanisms (Nicolás-Ávila et al., 2020). This protective role is exemplified in neurodegenerative pathologies and brain injury (Zhou et al., 2024; Hannah et al., 2024). Specifically, microglia transfer healthy mitochondria to compromised neurons via tunneling nanotubes (TNTs), which reduces oxidative stress and normalizes pathogenic gene expression. During acute brain injury, mitochondrial transfer further replenishes energy reserves while scavenging toxic reactive oxygen species (ROS), thereby establishing neuroprotection (Fu et al., 2025). Beyond endogenous processes, exogenous induction of mitochondrial transfer represents a promising therapeutic strategy for tissue repair. Osteoarthritis (OA) is a condition associated with this process, wherein mesenchymal stem cells (MSCs) transfer mitochondria to chondrocytes, thereby promoting their regeneration and repair (Fahey et al., 2022; Altuntaş et al., 2025). Interestingly, under different circumstances, the identities of the receptor and donor in mitochondrial transfer can be interchangeable (Nicolás-Ávila et al., 2020; Ikeda et al., 2021). In other words, certain cells may act as mitochondrial donors under specific conditions, and under other conditions, they may switch to become receptors. This suggests that many cells have the ability to both transfer and receive mitochondria, and this ability may be regulated. Furthermore, mitochondrial intercellular transfer involves not only the relationship between receptors and donors, but also potentially complex multicellular interactions, which warrant further investigation in future research.

**TABLE 1:** The role of mitochondria transfer in physiological functions and disease participation.

Position	Donor	Recipientor	Mitochondrial state	Function	Disease	Mitochondrial source	Description	Ref.
Epithelium								
	Platelets	MSC	Healthy	Promoting angiogenesis, wound healing and antimicrobial defence	Tissue damage/malnutrition	exogenous	interventions	Levoux et al. (2021)
Connective tissue								
Bone	Osteoblasts	Dendritic networks	Healthy	Supporting metabolism and bone mineral homeostasis	——	——	physiology	Gao et al. (2019)
	Osteoblasts	Bone progenitor cells	Healthy	Enhancing osteoblast differentiation	Bone damage	——	pathology	Feng et al. (2023)
	MSC	Chondrocytes	Healthy	Improvement of inflammation and stress stimulation	Osteoarthritis	exogenous	interventions	Wang et al. (2020), Fahey et al. (2022)
Adipose tissue	Brown adipocytes	Macrophages	Healthy	Regulation of adaptive thermogenesis	——	——	physiology	Rosina et al. (2022)
	Adipocytes	Macrophages	Healthy	Promoting immunity and support tissue homeostasis	Obesity	——	pathology	Brestoff et al. (2021)
	White adipocytes	Heart	Healthy	Protecting the heart from injury	Obesity	——	pathology	Ikeda et al. (2021)
Blood	Mitochondria	HSC	Healthy	Promoting haematopoiesis	Mitochondrial dysfunction	exogenous	interventions	Kawano et al. (2023)
	Mitochondria	HSC	Healthy	Promoting haematopoiesis	SLSMD	exogenous	interventions	Jacoby et al. (2022)
	BMSC	HSC	Healthy	Responding to bacterial infection	Infection	exogenous	interventions	Mistry et al. (2019)
	Mitochondria	Blood circulation	Healthy	Improving nerve function	Leigh syndrome	exogenous	interventions	Ritsuko et al. (2024)
Other	MSC	Macrophage cells	Healthy	Improving inflammation, damage repair	Diabetic kidney injury	exogenous	interventions	Yuan et al. (2021)
	MSC	Macrophage	Healthy	Improving inflammation, damage repair	Lung injury	exogenous	interventions	Morrison et al. (2017)
Muscular tissue								
	Cardiomyocytes	Macrophages	Damaged	Supporting cardiac homeostasis	——	——	physiology	Nicolás-Ávila et al. (2020)
	Mitochondrial exocysts	Cardiomyocytes	Healthy	Improving cardiac function after myocardial infarction	Myocardial infarction	autologous	interventions	Ikeda et al. (2021)
	PN-101 Mitochondria	Myocytes	Healthy	Anti-inflammatory effects	Refractory polymyositis	exogenous	interventions	NCT04976140
Neurology								
	Astrocytes	Neurons	Healthy	Supporting neuronal viability	Ischaemic stroke	——	pathology	Zhou et al. (2024)
	Macrophages	Neurons	Healthy	Limiting inflammatory pain signal propagation	Neuroinflammatory pain	——	pathology	van der Vlist et al. (2022)
	PN-101 Mitochondria	Dopaminergic neuronal cells	Healthy	Neuroprotective Effects	PD	exogenous	interventions	Eo et al. (2024)
	Neural stem cells	Macrophages	Healthy	Reducing expression of pro-inflammatory markers	MS	exogenous	interventions	Peruzzotti-Jametti et al. (2021)
	Mitochondria	Glial cells/neurons	Healthy	Attenuating glial cell proliferation and loss of hippocampal neurons	Hippocampal damage due to SE	exogenous	interventions	Jia et al. (2023)
	Mitochondria	Glial cells/neurons	Healthy	Increasing cell viability	Ischaemic stroke	autologous	interventions	Norat et al. (2023)
Cancer/Tumour								
	Cancer cells	Macrophages	Damaged	Improving mitochondrial metabolism, driving leukaemia transformation	Leukaemia	——	pathology	Weinhäuser et al. (2023)
	Macrophages	Cancer cells	Healthy	Accumulation of reactive oxygen species, promotion of proliferation	Cancer	——	pathology	Kidwell et al. (2023)
	T-cell	Cancer cells	Healthy	Weaking immune cell capacity, promotion of cancer cells proliferation	Cancer	——	pathology	Zhang et al. (2023b)
	Platelets	Cancer cells	Healthy	Promoting disease progression, anti-tumour function	Cancer	——	pathology	Lazar and Goldfinger (2021)
	BMSC	T-cells	Healthy	Enhancing T-cell metabolism and anti-tumour effects	Tumour	exogenous	interventions	Baldwin et al. (2024)
	Mitochondria	Cancer cells	Healthy	Altering metabolic pattern	Colon cancer	exogenous	interventions	Xu et al. (2021a)

MSC, mesenchymal stem cell; HSC, haematopoietic stem cells; SLSMD, single large-scale mitochondrial DNA, deletion; BMSC, bone marrow derived mesenchymal stem cells; PN-101, Mitochondria, human umbilical cord mesenchymal stem cell-derived mitochondria; PD, Parkinson’s disease; MS, multiple sclerosis; SE, status epilepticus.

In the senior population, OA is a prevalent joint disorder and plays a major role in causing disability. The mechanisms underlying OA are closely associated with mitochondrial function. A substantial body of research indicates that mitochondrial dysfunction results in energy depletion (Singh et al., 2010), the accumulation of ROS (Shi et al., 2025), variation in mtDNA (Mercedes et al.), and calcium metabolism (Wenyu et al., 2024). The initiation and advancement of OA are influenced by these combined factors (Sun et al., 2021; Durán-Sotuela et al., 2023; Blanco et al., 2018). For these reasons, targeted interventions aimed at mitochondrial function have emerged as a potential modifying therapy for OA. Such interventions primarily encompass drug-targeted regulation (Zhang Y. et al., 2023; Chen et al., 2022) and biological interventions using mesenchymal stem cells (Kim et al., 2022; Zhai et al., 2022). Artificial mitochondrial transfer has been shown to effectively treat experimental OA phenotypes in recent studies, establishing novel therapeutic avenues for OA (Kim et al., 2023; Lee et al., 2022). This review discusses the definitions and attributes of mitochondrial transfer and OA, as well as the established mitochondrial-related molecular mechanisms implicated OA pathogenesis. Furthermore, we will explore artificial mitochondrial transfer as a potential a therapeutic option for OA and propose pertinent scientific questions and research methodologies that warrant further investigation in the domain of mitochondrial therapy for OA.

## 2 The biological basis of mitochondrial transfer

In the early stages of mitochondrial research, it was widely believed that mitochondria within cells were solely inherited through vertical transmission during cell division or through mitochondrial biogenesis. However, in 2006, researchers observed the phenomenon of mitochondrial transfer between co-cultured cells, a process that could rescue ρ0 cells lacking mtDNA (88). Since then, mitochondrial transfer has been recognized as an important higher-order biological function of mitochondria, leading to increased attention and extensive investigation in the field. The biological mechanism of mitochondrial transfer is a highly complex and multi-layered process, involving various molecules, the cytoskeletal system, and intracellular dynamic processes. The biological structural foundation of mitochondrial transfer includes mitochondrial membrane proteins (Ahmad et al., 2014), motor proteins (Joshi et al., 2019), ATP generation and utilization (Spees et al., 2006), regulatory mechanisms, as well as the cytoskeleton and extracellular vesicles. The unique biological structure of mitochondria allows them to exhibit diversity in physiological processes and functions, enabling them to dynamically and reversibly adapt to energy demands, environmental changes, and other. These structures and mechanisms work in coordination to ensure that mitochondria can undergo dynamic transfer within cells as needed, in order to meet energy demands, respond to environmental changes, and support the normal functioning of the cell.

### 2.1 Mitochondrial membrane proteins and motor proteins

The outer and inner membranes of mitochondria are the core of their structure. They not only participate in maintaining mitochondrial functions (such as ATP synthesis, metabolite transport, etc.) but also play crucial roles during the transfer process (Hu et al., 2024; MacVicar et al., 2019; López-Doménech et al., 2018). Receptor proteins on the mitochondrial outer membrane interact with motor proteins in the cytoskeleton, helping mitochondria attach and move along the cytoskeleton on microtubules or microfilaments. Some proteins are located on the mitochondrial inner membrane and are involved in transport and positioning functions; they may regulate the stability and transfer of mitochondria by interacting with microtubules or microfilaments. Mitochondrial Rho GTPase 1 (Miro 1) is a mitochondrial-associated Rho-GTPase, a type of GTPase found on the mitochondrial outer membrane. The primary function of Miro one is to regulate the positioning and movement of mitochondria. During mitochondrial transfer, mitochondria use Miro one to shuttle along the actin-microtubule highways into the cytoplasm of the recipient cell (Ahmad et al., 2014).

Mitochondrial transfer relies on intracellular motor proteins, which drive the movement of mitochondria. Motor proteins typically move along the negative end of microtubules (toward the centrosome), pulling mitochondria from the cell membrane toward the cell center or other regions. On the other hand, dynein proteins move along the positive end of microtubules (toward the distal end), pushing mitochondria toward the cell membrane or protrusions. These motor proteins form stable complexes with mitochondrial membrane proteins, enabling mitochondria to move in a specific direction (López-Doménech et al., 2018). Studies have reported that mitochondrial quality control processes mediated by migration bodies involve the regulation of motor proteins. When mitochondria are exposed to mild stress, damaged mitochondria are transported to migration bodies, where they are subsequently expelled from the migrating cells (Jiao et al., 2021). Research has also shown that microglial cells rely on dynamin-related protein 1 (DRP1) and mitochondrial fission protein 1 (FIS1) to release free mitochondria. This indicates that both the movement of mitochondria within the cell and their quality control during stress responses are tightly regulated by motor proteins, ensuring proper mitochondrial distribution and function in the cell (Joshi et al., 2019).

### 2.2 ATP generation and utilization

**FIGURE 1:**
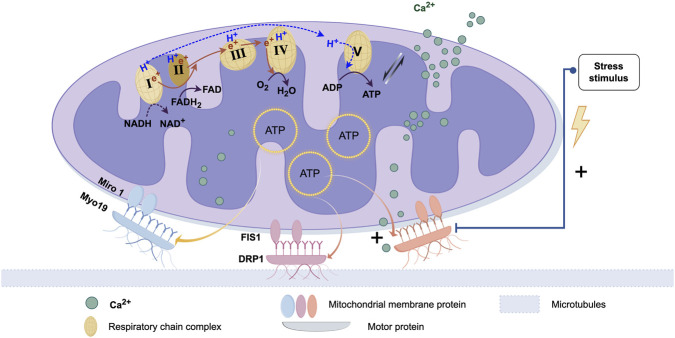
Proteins associated with mitochondrial transfer and regulatory processes. The biological basis of mitochondrial transfer lies in the pairing and binding of membrane proteins and dynamin proteins. After protein binding, movement is typically facilitated by microtubules. The energy for this movement primarily comes from ATP produced by the mitochondria. This process is also regulated by calcium ion uptake and bio-mechanical stress stimuli. (Graphic is created by FigDraw. Copyright Code:ORURW7e3c7).

All mitochondria possess a core OXPHOS system, which consists of five multi-protein complexes, with four being part of the electron transport chain (ETC.). On the inner membrane of the mitochondria, the free energy (ΔG) generated by these complexes establishes an electrochemical gradient, which in turn creates a membrane potential, serving as a major source of energy (Edman et al., 2024). The formation of membrane potential is not only essential for maintaining mitochondrial permeability, channel integrity, and regulating the morphology of mitochondrial cristae, as well as mitochondrial fusion/fission dynamics, but also drives several other mitochondrial functions. These include ion uptake (such as Ca^2+^, Na^+^, and Mn^2+^), synthesis of nuclear-encoded proteins and antioxidant defense regenerated, precursor Fe/S clusters, antiviral signaling, and ATP synthesis by the Fo ATP synthase F1 subunit, among others. ATP also powers motor proteins (such as dynein and kinesin), driving the transfer of mitochondria, and ultimately serves as the energy source for the recipient cell to uptake mitochondria (Figure 1) (Spees et al., 2006).

### 2.3 Regulation of mitochondrial transfer

Mitochondrial transfer is not only dependent on the action of motor proteins but is also regulated by various signaling pathways. The regulation of mitochondrial transfer involves calcium ion (Ca^2+^) uptake (Wang and Schwarz, 2009), cellular stress responses (Wang et al., 2018), and the stability of microtubules. Changes in the Ca^2+^ concentration can affect the position and movement of mitochondria (Emily et al., 2018), especially in neurons, where calcium signaling is crucial for the transfer of mitochondria to and from the synaptic sites (Hayakawa et al., 2016). When cells encounter stress (such as hypoxia (Hu et al., 2023), oxidative stress (Wang et al., 2018), etc.), mitochondria may alter their position or accumulate in specific regions to adapt to environmental changes. The stability and arrangement of the microtubules inside the cell directly influence the movement of mitochondria. Mitochondrial morphology changes, such as fission and fusion, also play a major role in transfer process (Valm et al., 2017). Mitochondrial fission typically occurs when the cell needs to regulate mitochondrial number (for example, during cell division) (Jiao et al., 2021), while fusion helps mitochondria merge into a more powerful functional unit (Nasoni et al., 2021). These morphological changes are closely related to the cell’s energy needs and also affect the distribution and transfer of mitochondria within the cell (Fu et al., 2025).

### 2.4 Cytoskeleton and extracellular vesicles

**FIGURE 2:**
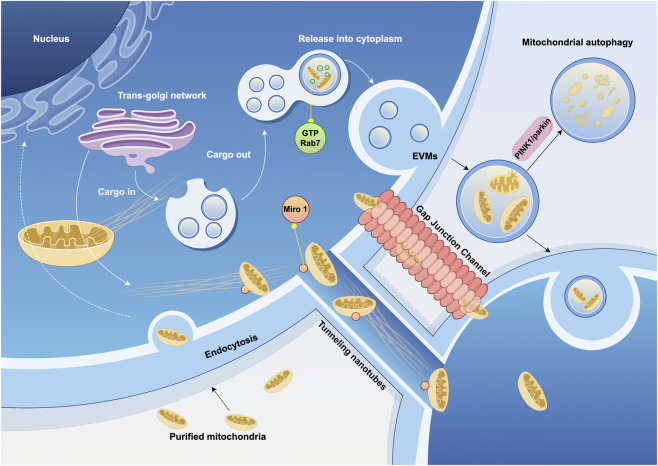
Cytoskeleton and extracellular vesicles associated with mitochondrial transfer. Mitochondrial transfer utilizes microtubules as “tracks” to move mitochondria to specific compartments through the pairing of specialized mitochondrial membrane proteins and dynamin proteins. Mitochondria can also be transported loaded into vesicles and released extracellularly under the regulation of GTPases. The ultimate fate of extracellular vesicles derived from mitochondria—whether they are internalized, released, or trigger autophagy—is also regulated by signaling pathways. EVMs, extracellular vesicles contain mature mitochondria. (Graphic is created by FigDraw. Copyright Code: SSWIW00a0a).

Mitochondrial transfer between cells occurs in various tissues *in vivo* and can be classified into mediated and unmediated mechanisms. Mediated transfer occurs through transient cell connections, based on the cytoskeletal system, which provides “tracks” and “power” for mitochondrial movement (Boldogh and Pon, 2007). Cells establish connection channels, which are elongated, membrane-wrapped structures that allow organelles to transfer between cells. Mitochondria are transferred from 1 cell to another through these connections (Figure 2). These cell connections are typically formed by TNTs and/or gap junction channels (GJC) mediated by connexin 43 (Cx43). TNTs are dynamic structures that permit the exchange of proteins, soluble molecules, and organelles. The transfer of organelles within TNTs is crucial for regulating cell growth, signaling, and disease progression (Nasoni et al., 2021; Saha et al., 2022). Cx43 exists in the mitochondrial inner membrane in a hemichannel form, is related to gap junction channels, and may also regulate the formation of TNTs (Tishchenko et al., 2020).

Extracellular vesicle (EV) can also serve as medium for mitochondrial transfer, with mature mitochondria present in the vesicles secreted by cells, which are then delivered to recipient cells. Mitochondrial-derived vesicles (MDVs) have been found in a variety of cell types, including osteoblasts (Suh et al., 2023), bone marrow adipocytes (Crewe et al., 2021), and cardiomyocytes (Ikeda et al., 2021), as well as in migrating cells (Duan et al., 2020). In the extracellular vesicles of these cells, damaged mitochondria are more commonly observed. These structures may be regulated by the PINK1/parkin pathway. Parkin is an E3 ubiquitin ligase that binds PINK1, marking damaged mitochondria for degradation by mitophagy. In the absence of parkin, this elimination process is impaired (Ikeda et al., 2021). Rab7, a small GTPase, plays a critical role in regulating the maturation of endosomes. When extracellular vesicles contain mature mitochondria (EVMs), GTP-bound Rab7 is the determinant of EVM secretion out of the cell (Liang et al., 2023).

Mitochondrial transfer can also occur in a free form. One way is through direct cell fusion, which results in the mixing of cytoplasm and organelles. Another way is through direct contact, where mitochondria are transferred via direct contact between cell membranes, and the mitochondria are released into the extracellular space in a free state, with recipient cells actively capturing these mitochondria (Brestoff et al., 2021). This free transfer is more commonly observed in blood components, where free mitochondria, lacking extracellular vesicles, are approximately 0.5–1 μm in diameter and comprise the full-length mtDNA genome. However, their origin is heterogeneous, suggesting that free mitochondria in blood may originate from different cell types (Boudreau et al., 2014; Borcherding et al., 2022).

## 3 Overview of the characteristics of OA

### 3.1 Clinical features and pathological process of OA

OA is a leading cause of musculoskeletal pain and disability and is a common type of arthritis. As a chronic inflammatory condition associated with the aging process, OA primarily affects individuals aged 65 and older, with its prevalence significantly increasing with advancing age (Motta et al., 2023). Clinically, OA is characterized by symptoms such as joint stiffness, chronic pain, instability, deformity, radiographic evidence of joint space narrowing (Heidari, 2011). Given the complexity of its pathogenesis, current treatment primarily focus on alleviating joint stiffness and pain, aiming to provide palliative symptom relief and improve the quality of life for affected individuals.

The onset of this disease is attributed to an active dynamic imbalance between repairing and destroying joint tissue, rather than the passive degenerative process or mere wear-and-tear typically described (Prieto-Alhambra et al., 2014; Fu et al., 2018). And then, alterations occur in the components of cartilage, resulting in the loss of its integrity (Loeser et al., 2016). These compositional changes alter the properties of the cartilage and increase its susceptibility to physical damage. Initial erosion usually occurs at the surface, followed by deeper fissures in the cartilage and subsequent expansion of calcified cartilage areas. During the repair process, hypertrophied chondrocytes demonstrate enhanced synthetic activity, leading to the production of matrix degradation byproducts and pro-inflammatory mediators. These factors disrupt the regulation of chondrocyte function and influence the adjacent synovium, stimulating both proliferation and inflammatory responses. The proliferating synovial cells also release pro-inflammatory products, a process that is accompanied by tissue hypertrophy and increased vascular distribution. Additionally, in the subchondral bone, there is an elevation in bone turnover and vascular invasion, which facilitates the extension from subchondral bone into the cartilage. Furthermore, the formation of bone spurs at the joint margins due to endochondral ossification are significantly influenced by inflammatory biological factors, as well as by mechanical overload and abnormal joint kinematics (Hunter and Bierma-Zeinstra, 2019). Collectively, these factors disrupt the dynamic balance of cartilage, leading to an increase in stiffness and changes in the biological properties, ultimately resulting in the development of OA.

### 3.2 Etiological analysis of OA

Among the etiologies of OA, limited cartilage regeneration is a key factor in disease progression. Articular cartilage is a thin layer of tissue that covers the surfaces of articulating bones, typically measuring between 2 and 4 mm in thickness, and is referred to as “hyaline cartilage” due to its clear and transparent appearance (Fujii et al., 2022). This type of cartilage exhibits considerable elasticity and lubricity, enabling it to effectively support mechanical loads and facilitate smooth movement between bones, thereby significantly reducing friction during joint activities (Fujii et al., 2022; Iwamoto et al., 2013). However, the regenerative capacity of cartilage is relatively limited and is influenced by various factors. Consequently, self-repair following injury often proves inadequate, which can lead to the development of degenerative diseases, particularly OA (123).The academic community has proposed several hypotheses to explain the failure of cartilage regeneration. These hypotheses include the scarcity of regenerative potential cells, pathological mechanical alterations, persistent inflammation, and metabolic stress, among others.

It is important to note that the physiological characteristics of chondrocytes significantly influence their regenerative capacity. The low density of chondrocytes, coupled with the relatively limited proliferative ability of mature chondrocytes, is considered the main reason for the insufficient self-regeneration capacity of cartilage (Kai-di et al., 2021; Jerry and Kyriacos, 2004). Additionally, aging is associated with pronounced senescence in chondrocytes, resulting in a reduction in cell density and *in vitro* proliferative capacity, a phenomenon that is particularly evident in patients with post-traumatic OA (Barbero et al., 2004; David et al., 2017). Pathological mechanical changes within the joint can lead to cartilage loss. Trauma or chronic degenerative changes may modify the load distribution across the joint, thereby affecting the contact area of the load. This mechanical alteration may stimulate the activation of osteoclasts, fibroblasts, and macrophages, which release pro-inflammatory mediators that gradually degrade the ECM, ultimately resulting in damage to the collagen network and inflammation of the synovium (Caravaggi et al., 2021; Assirelli et al., 2022). The widely studied concept of “mechanical inflammation” refers to the pathological process of inflammatory signaling caused by mechanical stress. Meanwhile, non-resolving inflammation may result in histological changes in cartilage. Inflammatory stress impairs the activity and matrix synthesis of chondrocytes and promotes matrix degradation by stimulating the production of various matrix metalloproteinases and interleukin-2 (Andriacchi et al., 2004).

Moreover, chondrocytes depend on molecular mechanisms that are specifically adapted to low-oxygen and low-nutrient environments under physiological conditions for normal operation. Following an injury, it becomes difficult to maintain the local partial oxygen pressure at an ideal level, leading to increased energy demands that can affect the functionality of chondrocytes and the requisite microenvironment. At this point, cells experience metabolic changes, particularly the dysregulation of the glycolytic pathway, resulting in the accumulation of lactic acid, which further alters the local microenvironment (Dolzani et al., 2019). This metabolic change inhibit matrix synthesis and accelerates the degradation process of cartilage.

## 4 Mitochondrial dysfunction in OA

OA is a heterogeneous disease with various potential pathogenic pathways. Each common risk factor for OA may trigger different mechanisms of disease, particularly those involving oxidative stress, impaired biosynthesis and growth responses of chondrocytes and osteoblasts, synovial inflammation, and increased degradation and calcification of the cartilage matrix (Mathiessen and Conaghan, 2017; Lepetsos and Papavassiliou, 2016; Blanco et al., 2011). OA is also associated with phenotypic differences, such as age and obesity, and the pathogenesis of OA varies markedly from one population to the next, with possible interactions between the mechanisms (Muthu et al., 2023). In this context, OA can be viewed as a syndrome rather than a singular disease, with potential interactions among its mechanisms that remain to be fully elucidated. Currently, there are no clear classification standards for mitochondrial dysfunction in OA. To describe the changes occurring in mitochondria within OA more clearly, this article categorizes them into three distinct categories: structural abnormalities, alterations in biological function, and biological interactions.

### 4.1 Structural abnormalities

When mitochondrial homeostasis or mitosis is impaired, dysfunctional mitochondria cannot be removed in a timely manner, leading to a disturbance in the dynamic balance of mitochondria and further affecting chondrocyte health. The regulatory mechanisms of mitochondrial quality control are impaired in individuals diagnosed with OA. This damage involves mitochondrial biogenesis, mitophagy, and mitochondrial dynamics, which include processes of fission and fusion (Guo et al., 2025). Observations have shown that mtDNA in patients with OA exhibits damage and mutations, resulting in a decrease in the size, quantity, and overall content of mitochondria (Alejandro et al., 2024; Morena et al., 2022). Genomic instability can cause damage to mtDNA, further affecting mitochondrial function.

Damaged mtDNA can cause mitochondrial respiratory chain to malfunction, subsequently resulting in the production of ROS. Excessive production of ROS is a prominent feature of mitochondrial dysfunction. It can cause diverse forms of mtDNA damage. The phenomenon encompasses a range of structural alterations, including oxidative base damage, strand breaks, and telomere shortening. It is hypothesised that such damage may ultimately result in cell cycle arrest and cellular senescence (Macip et al., 2002). In addition, research findings have indicated that particular mtDNA variants, encompassing specific haplotypes, exhibit a notable correlation with the initiation and progression of OA. (Vermulst et al., 2007). The relationship between mtDNA haplotypes and OA may exhibit heterogeneity across different geographic populations, indicating that these haplotypes could serve as potential biomarkers for the diagnosis and prognosis of OA (Fang et al., 2014).

Mitochondrial dysfunction can induce alterations in autophagy, which may lead to the accumulation of defective mitochondria, thereby potentially increasing mitochondrial levels in chondrocytes of patients with OA. Furthermore, chronic oxidative damage may result in a reduction of FIS1, which promotes mitochondrial elongation and the formation of giant mitochondria (Lee et al., 2007). This elongation may contribute to the excessive expansion of the mitochondrial network, which plays a significant role in the progression of age-related diseases such as OA.

The mitochondrial permeability transition pore (mPTP) comprises subunits that are situated within both the inner and outer mitochondrial membranes (Bonora et al., 2022). In the chondrocytes of OA patients, factors such as oxidative stress, calcium overload, and depolarization of the inner membrane may lead to the pathological opening of mPTP. The transient activation of mPTP, triggered by a variety of stimuli, can induce protective pathways, a phenomenon referred to as “mitochondrial excitability.” Conversely, a persistent opening of mPTP can culminate in the release of apoptotic factors, which, if prolonged and unregulated, may ultimately lead to the demise of the cartilage cells (Patel et al., 2021).

### 4.2 Alterations in biological function

**FIGURE 3:**
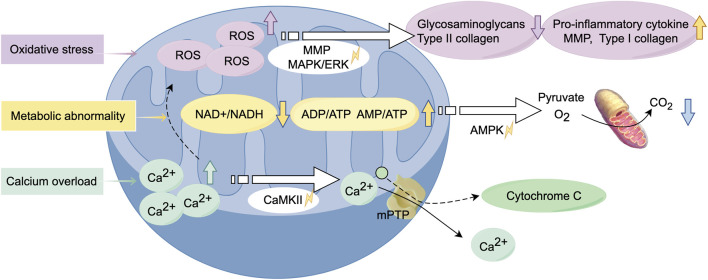
Alterations in biological function of mitochondria during the development of osteoarthritis. Mitochondrial dysfunction leads to oxidative stress, resulting in the excessive production of ROS. This process activates relevant signaling pathways, triggering inflammatory responses and metabolic abnormalities. Calcium overload exacerbates this process, causing alterations in mitochondrial membrane permeability, ultimately leading to chondrocyte cell death. (Graphic is created by FigDraw. Copyright Code: SSTIY92b49).

Oxidative stress-mediated mitochondrial dysfunction in chondrocytes constitutes a key driver of OA pathogenesis (Figure 3). ROS are widely recognized as contributors to chondrocyte damage and the progression of OA. The excessive production of ROS disrupts joint homeostasis, triggering cell apoptosis and accelerating metabolic degradation, thereby impairing various structures of the joint (Loeser et al., 2016). Interleukin-1 beta (IL-1β) and tumor necrosis factor-alpha (TNF-α) are major inflammatory mediators in the pathogenesis of OA, and their actions are closely related to mitochondrial dysfunction (van Vulpen et al., 2015). Oxidative stress is primarily attributed to dysfunctional mitochondria, which generate ROS such as superoxide anions and hydroxyl radicals. Excessive ROS has been observed to induce an inflammatory response, which has been linked to the expression of matrix metalloproteinases (MMPs) and the activation of associated signalling pathways, including the MAPK/ERK pathway. The aforementioned alterations have been shown to impede the process of glycosaminoglycan and type II collagen synthesis, whilst concurrently inducing an escalation in the concentrations of both type I collagen and MMPs. Furthermore, there is a concomitant increase in pro-inflammatory cytokine production (Rieder et al., 2018). Furthermore, excessive ROS can also damage mtDNA and reduce its repair capacity, highlighting the complex relationship between mitochondrial dysfunction and OA, making it a potential therapeutic target. In a rat model of OA, a drug delivery system (DDS) containing melatonin was administered via intra-articular injection. Melatonin was found to mitigate cartilage degeneration through its antioxidant properties, which indirectly enhanced mitochondrial function and facilitated the repair of the cartilage matrix (Zhang Y. et al., 2022).

Damaged mitochondria lead to insufficient energy production, producing impaired energy metabolism and an inability to maintain normal cellular physiological functions, thereby exacerbating chondrocyte damage and apoptosis. In response to environmental stress, chondrocytes exhibit a tendency to switch between different metabolic pathways to adapt to these changes (Lee et al., 2012). The alterations in metabolic pathways are closely related to mitochondrial dysfunction, which may result in a marked decrease in the NAD+/NADH equilibrium, an increased rate of anaerobic glycolysis, and alterations in lipid and amino acid metabolism (Stein and Imai, 2012). The decline in the NAD+/NADH equilibrium, in combination with an increase in the ADP/ATP and AMP/ATP ratios, leads to the activation of AMP-activated protein kinase (AMPK). The balance between OXPHOS and glycolysis is predominantly regulated by the AMPK and mTOR signaling pathways (Herzig and Shaw, 2018). Changes in the NAD + pathway can severely impact mitochondrial function, potentially leading to cell growth arrest or impairing cellular metabolism through the depletion of sirtuins (SIRT) 3 and SIRT 5 (Miwa et al., 2022). It has been reported to improve respiratory chain function through the induction of SIRT3, with particular emphasis on its potential application in the treatment of OA (Zhang Y. et al., 2023).

Abnormal calcium regulation leads to overproduction of reactive oxygen species, mitochondrial depolarisation and reduced mitochondrial membrane potential, which further contributes to chondrocyte apoptosis. In the context of OA, the elevation of cytosolic Ca^2+^ concentration prompts mitochondria to rapidly absorb Ca^2+^, thereby preventing calcium overload within the cells (Bravo-Sagua et al., 2017). However, excessive calcium accumulation within mitochondria can result in heightened production of ROS, leading to mitochondrial dysfunction and senescence. This phenomenon has the capacity to exert a deleterious effect on various facets of mitochondrial metabolism, including nitric oxide synthesis, the process of releasing cytochrome c, the process of altering mitochondrial membrane permeability and the process of activation of calcium/calmodulin-dependent protein kinase II (CaMKII) signalling pathways (Brookes et al., 2004; Peng and Jou, 2010). In response to this pathological alteration, the utilization of nanoparticles carrying siRNA alleviates mitochondrial calcium overload in MSCs, thereby regulating dysfunctional mitochondrial autophagy. This approach has demonstrated efficacy in the treatment of OA (Zhai et al., 2022).

In an environment characterized by oxidative stress, chondrocytes may undergo regenerative mitochondrial transfer. Research has observed mitochondrial transfer to chondrocytes through fluorescence imaging, finding that this process is associated with enhanced mitochondrial function, likely correlated with the upregulation of gap junction protein Cx43 expression (Wang et al., 2021; Mayan et al., 2015). In the context of an inflammatory environment, the transfer of mitochondria from MSCs to chondrocytes is significantly increased, with Cx43 playing a critical mediating role in this process (Irwin et al., 2024). Upon contact with damaged cartilage, MSCs localize to the injury site and deliver mitochondria to chondrocytes through cellular protrusions (Fahey et al., 2022). Experimental results indicate that combined inflammatory and mechanical stress enhances mitochondrial transfer between chondrocytes, suggesting that mitochondrial transfer may occur spontaneously in the progression of OA. However, further experimental validation is necessary to substantiate these observations.

### 4.3 Biological interactions

**FIGURE 4:**
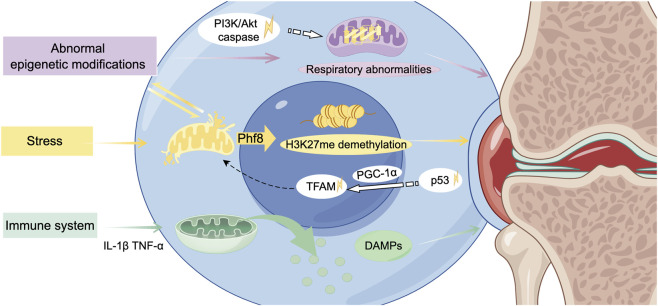
Mitochondria-related biological interactions in the development of osteoarthritis. Under biomechanical stress, mitochondrial dysfunction interacts with aberrant epigenetic modifications, contributing to alterations and damage in the chondrocyte nucleus. The subsequent release of release danger-associated molecular patterns (DAMPs) exacerbates the inflammatory response, ultimately driving the progression of osteoarthritis. TFAM, nuclear-encoded mitochondrial transcription factor A. (Graphic is created by FigDraw. Copyright Code: SSTIY92b49).

Chondrocytes have been observed to exhibit a response to mechanical stimuli, which is mediated by the extracellular matrix (ECM). The ECM plays a crucial role in the regulation of the epigenetics in diseases. Mitochondria may serve as a potential communication medium between mechanical signals and epigenetics modifications (Figure 4). The relationship between inhibition of matrix synthesis and activation of matrix degradation is also affected in OA. Redox abnormalities in the mitochondria have been shown to inhibite matrix synthesis whilst concomitantly activating matrix metalloproteinases, thus promoting the degradation of matrix components and, by extension, degenerative cartilage lesions. Within the context of OA, an elevated level of cartilage matrix rigidity in regions that are subjected to substantial stress has been observed to result in considerable impairment to the mitochondria of the cartilage cells. This, in turn, initiates the demethylation of histone H3 lysine 27 trimethylation (H3K27me3). The opening of the mPTP promotes the translocation of plant homeodomain finger protein 8 (Phf8) to the nucleus, catalyzing the demethylation of H3K27me3 (Kan et al., 2024).

Abnormal epigenetic modifications are considered preliminary factors in the occurrence and development of OA, with a strong correlation to mitochondrial dysfunction (Jiang et al., 2024). Genetic and epigenetic abnormalities have been demonstrated to result in increased mitochondrial respiration and glycolysis, elevated levels of free radicals and pro-inflammatory cytokines, and augmented rates of apoptosis. Research has demonstrated that exposure to a hyperglycemic environment *in utero* increases the vulnerability to OA in later adulthood, attributable to persistently diminished expression levels of Sirt3. The downregulation of Sirt3 results in impaired mitotic function of the cells responsible for cartilage production (chondrocytes), abnormal mitochondrial respiration, and the incapacity to efficiently eliminate aged and damaged mitochondria in a timely manner. The consequence of this is an imbalance in mitochondrial homeostasis, which in turn triggers disease through epigenetic regulation (Li et al., 2024a).

During the occurrence of OA, dysfunctional mitochondria induce the production of cytokines. These cytokines further promote chondrocyte apoptosis and accelerate the progression of OA. In the initial stages, it has been observed that both the Phosphatidylinositol 3 kinase (PI3K)/Protein kinase B (Akt) signalling pathway (relevant to cartilage cell death) and the caspase pathway (pertinentto the initiation of cell death) are activated. This results in oxidative stress-induced cartilage cell death, a process involving the mitochondria (Li et al., 2019). These signaling cascades are crucial for various biological processes, and any imbalances within these pathways can disrupt normal biological processes and lead to diseases. Additionally, this dysregulation may activate the tumor protein p53 signaling pathway, inhibiting peroxisome proliferator-activated receptor-γ coactivator-1α (PGC-1α) and peroxisome proliferator-activated receptor gamma co-activator 1β (PGC-1β). This process may contribute to the exacerbation of mitochondrial dysfunction (Sahin et al., 2011).

During the process known as mitochondrial biogenesis, the nuclear-encoded mitochondrial transcription factor A (TFAM) is an essential component of mtDNA transcription and the regulation of mitochondrial biogenesis (Picca and Lezza, 2015). PGC-1α is activated by physiological stressors, which in turn activate transcription factors and regulate the transcription of TFAM (Wu et al., 1999). Furthermore, PGC-1α can also modulate the activity of other transcription factors such as peroxisome proliferator-activated receptor γ (PPARγ), Yin Yang 1(YY-1), and GA (Guanine-Adenine)binding protein transcription factor (GABPA), thereby impacting mitochondrial function (Scarpulla, 2011). The intravenous administration of meta-Defensomes polymer nanoparticles in collagenase-induced OA mice resulted in the transformation of pro-inflammatory classically activated macrophages (M1 macrophages) into anti-inflammatory alternatively activated macrophages (M2 phenotypes). This intervention led to an increase in the expression of mitochondrial transcription factor A, restoration of aerobic respiration, and a significant reduction in synovitis, thereby effectively inhibiting the early progression of OA (Zhang L. et al., 2022).

The relationship between mitochondrial dysfunction and the activation of the immune system is complex and intertwined, serving as a significant factor in the pathogenesis of OA. Dysfunctional mitochondria release danger-associated molecular patterns (DAMPs), which can trigger innate immune responses and induce inflammation (Miwa et al., 2022; Wan et al., 2014). As the human organism ages, there is an observed increase in the concentration of free mtDNA within cells. This increase has been shown to correlate with the presence of markers associated with sterile inflammation. This increase activates immune response pathways, including the NOD-like receptor family pyrin domain containing 3 (NLRP3) inflammasome, which belongs to the family of nucleotide-binding oligomerization domain-like receptors. The activation of the NLRP3 inflammasome consequently results in the maturation of the second messenger GMP-AMP, which in turn activates the immune response, thereby promoting inflammatory responses (White et al., 2014).

## 5 Practice and prospects of mitochondrial transfer involvement in OA therapy

### 5.1 Mitochondrial transfer in OA therapy

In the management of OA, in addition to conventional symptomatic treatments and surgical interventions, disease-modifying therapy (DMT) is increasingly receiving scholarly attention (Li Z. et al., 2024). As a precision intervention, DMT corrects core OA etiologies through biomarker-directed pathway modulation, not only arresting disease trajectory but also activating endogenous chondrocyte regenerative mechanisms (Yulong et al., 2021; Ronghui et al., 2024; Giuliana et al., 2023; Kai et al., 2024).

Presently, numerous clinical trials in 2–4 Phase are in progress (https://www.clinicaltrials.gov/), with mesenchymal stem cell therapy and blood-derived therapies emerging as significant areas of research (Naomasa et al., 2017; Yong Sang et al., 2015). The sources of mesenchymal stem utilized in these therapies primarily include adipose-derived mesenchymal stem cells (Chen et al., 2021), bone marrow-derived mesenchymal stem cells (Vives et al., 2015; Orozco et al., 2014), umbilical cord-derived mesenchymal stem (Lim et al., 2021) and other types of MSCs(NCT03866330,NCT02037204). In these therapeutic modalities, MSCs are believed to ameliorate mitochondrial dysfunction in chondrocytes through mitochondrial transfer, thereby providing substantial foundational research support for their clinical application (Fahey et al., 2022; Angela et al., 2024). Recent investigations have identified a significant relationship between Cx43 and its truncated isoform gap junction protein alpha 1–20 kDa (GJA1-20k) in the transfer of mitochondria between highly oxidative cells. The overexpression of GJA1-20k in MSCs has been shown to enhance the transfer of mitochondria to chondrocytes, thereby facilitating the repair of cartilage tissue affected by OA (156). Consequently, the direct transfer of mitochondria to diseased joints emerges as a key facilitator.

Blood-derived treatment strategies predominantly encompass stromal vascular fraction (SVF) (Krześniak et al., 2021)and platelet-rich plasma (PRP) (Mayoly et al., 2019). PRP stimulates cell proliferation and reduces inflammation, oxidative stress and chondrocyte senescence. A related clinical trial in Phase 4 (NCT05660824) is a multicenter, parallel-group, triple-blind trial enrolling 130 patients. The aim of the study is to evaluate the clinical efficacy of SVF as an adjunctive therapy to PRP for functional and tissue regeneration in OA. PRP contain various organelles, including mitochondria, lysosomes, dense granules, and alpha granules (Zhai et al., 2022). After intra-articular injection, mitochondria in platelets are transferred to damaged chondrocytes, promoting chondrocyte repair and regeneration, which is also an effective treatment option for OA.

### 5.2 Current status of research on mitochondrial artificial transfer in OA therapy

Based on the complexity of mitochondrial dysfunction in OA, research conducted on 450 participants in the Osteoarthritis Initiative (OAI) study has demonstrated that indicators associated with mitochondrial dysfunction are linked to mtDNA variants and the risk of rapid progression of knee OA (80). This evidence supports the potential for mitochondrial-targeted therapies in the management of OA. Mitochondrial artificial transfer, which entails the supplementation of healthy and functionally normal mitochondria, represents a novel and effective therapeutic strategy for degenerative diseases related to bones and cartilage. Currently, many studies are exploring the artificial mitochondria transfer from other environments or cell types to treat various diseases (Ritsuko et al., 2024; Vijith et al., 2024). It is also known as mitochondrial transplantation (Caicedo et al., 2024; Christoph et al., 2022).

In the study of artificial mitochondrial transfer, various methods have been implemented, which can be primarily categorized into prenatal and postnatal artificial mitochondrial transfer. Early prenatal artificial mitochondrial transfer treatments have utilized ooplasm as a mitochondrial carrier, where healthy donor oocyte mitochondria and their mtDNA are directly injected into a recipient oocyte with pathogenic mtDNA mutations through micromanipulation (Barritt et al., 2001). The resultant cells contain both donor and recipient mtDNA and are subsequently utilized for *in vitro* fertilization. Recently, there has been a growing inclination towards utilizing stem cell-derived mitochondria in mitochondrial-assisted *in vitro* fertilization treatments. Mitochondria are extracted and purified from autologous ovarian stem cells (Labarta et al., 2019), autologous bone marrow stem cells (NCT03639506), or autologous urine-derived stem cells (NCT06020742), and are then co-injected with sperm into oocytes to improve embryo quality. Presently, several of these therapeutic strategies have progressed into the early clinical research.

Postnatal artificial mitochondrial transfer represents another area of exploration (Mitch, 2022). This therapeutic approach is based on the ability of exogenous mitochondria to enter cells. Mitochondria may be directly administered to patients following purification (Ying-Ting et al., 2019), or alternatively, the purified mitochondria can be incorporated into autologous cells prior to injection into the patient (Jacoby et al., 2022). Furthermore, purified mitochondria can be directly given to patients (Ikeda et al., 2021). In the treatment of OA, several experiments have been reported in animal experiments using autologous mitochondria and exogenous mitochondria, using intra-articular injections, to improve osteoarthritic symptoms and slow down the course of OA in various ways (Table 2). The results confirm the favourable biosafety of mitochondrial transfer following intra-articular injection in mammals. Mitochondrial injections are more biocompatible than cellular level modification treatments. Therefore, it may be more efficient to introduce purified, highly concentrated mitochondria into the lesion site artificially without the aid of exogenous cellular mediators (cells, plasma, etc.) to improve the therapeutic effect.

**TABLE 2:** Current studies applying purified mitochondrial transfer for osteoarthritis treatment.

Donor	Structure	Recipient	Targeted tissue	Mechanism of action	Treatment	Ref.
Homo sapiens	UC-MSCs-derived MT	collagenase-induced OA mouse model (12-week-old C57BL6 male mice)	left knee joint	Balanced cell redox, energy and metabolic homeostasis in the osteoarthritic chondrocyte preserving cartilage integrity	Intra-articular injection	Angela et al. (2024)
Homo sapiens	FMCs, MSCs-derived MT	MIA-induced OA mouse model (8-week-old Balb/c nude mice)	knee joint	A highly effective and promising strategy for delivery of MT to promote cartilage regeneration	Intra-articular injection based on membrane fusion	Kim et al. (2023)
Equus ferus caballus	healthy equine platelets-derived MT	adult horses	left equine intercarpal joint	Autologous mitochondria injection was well tolerated, providing essential safety information for clinical application in horses and humans	Intra-articular injection	Cassano et al. (2023)
Rattus norvegicus	BMSCs-derived MT	collagenase-induced OA rat model (4-6-week-old male SD rats)	right knee joint	Ameliorated pathological cartilage injury, suppressed inflammation, inhibited chondrocytes apoptosis, and improved osteoarthritis phenotype	Intra-articular injection	Yu et al. (2022)
Rattus norvegicus	L6 cells-derived MT	MIA-induced OA rat model (7-week-old male Wistar rats)	right knee joint	Improved mitochondrial function in chondrocytes indicated by membrane potential and oxygen consumption rate	Intra-articular injection	Lee et al. (2022)

UC, umbilical cord; MSCs, mesenchymal stem cells; MT, mitochondria; OA, osteoarthritis; FMCs, Fusogenic mitochondrial capsules; MIA, monoiodoacetate; BMSCs, bone marrow derived mesenchymal stem cells.

### 5.3 Prospects for mitochondrial transfer in the treatment of OA

**FIGURE 5:**
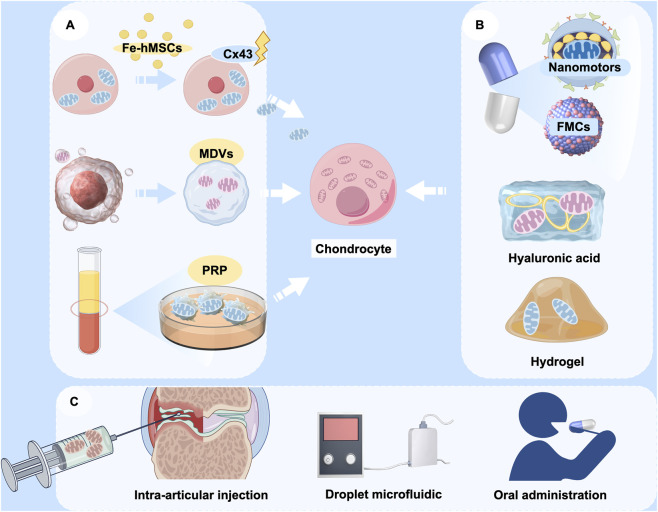
The potential application of mitochondrial transfer in OA therapy. **(A)** Various sources of donor mitochondria, including mesenchymal stem cells, mitochondrial-derived vesicles (MDVs), and platelet-rich plasma (PRP); **(B)** Encapsulation of therapeutic mitochondria with various delivery vehicles, including nanomotors, fusogenic mitochondrial capsules (FMCs), hyaluronic acid, and hydrogels; **(C)** Modes of cargo delivery to target cells, including intra-articular injection, droplet microfluidics, and oral nanoparticle capsules. (Graphic is created by FigDraw. Copyright Code: UYUWA7822c).

The application of the artificial transfer method of mitochondria is a very promising therapeutic strategy for the treatment of OA because of the obvious advantages of this method. However, mitochondria is large size about 0.5–1 μm and the efficiency of delivery by conventional endocytosis is very low, thereby constraining their clinical applicability (Sun et al., 2019). This limitation has prompted recent research to concentrate on optimizing the efficiency of mitochondrial transfer, primarily through two avenues: optimizing carriers and augmenting transfer efficiency (Figure 5). In addition to conventional stem cells, it has been identified that mitochondria can adapt to form structures known as mitochondrial-derived vesicles (MDVs), which exhibit repair functions (Min et al., 2025; Reut et al., 2023). Exosomes from adipose-derived mesenchymal stem cells (AdMSC-Exos) has been found to effectively donate mitochondrial components and improve macrophage mitochondrial integrity and OXPHOS levels. This restores metabolic and immune homeostasis of airway macrophages and attenuates inflammatory lung pathology (Miriam et al., 2021). Therefore, utilizing exosomes as carriers for mitochondrial transfer is supported by a physiological basis for therapeutic interventions.

Advancements in bioengineering have led to increased diversity and efficiency in the design of biological carriers. Fusogenic mitochondrial capsules (FMCs), which are composed of various lipids and liposomes, facilitate the rapid and efficient transport of mitochondria to chondrocytes, thereby promoting cartilage regeneration (Kim et al., 2023). Besides liposomes, hyaluronic acid has been employed in conventional carrier systems. Liposomes that are coated with hyaluronic acid have the capability to co-deliver the natural cyclic peptide RA-XII and mitochondrial-targeting photosensitizers, exhibiting high selectivity and potential for precise combination therapy in colorectal cancer (Xu et al., 2021b). Targeted modification of mitochondria is also an effective approach. Nanomotorized mitochondria has recently been developed with chemotactic targeting ability for damaged heart tissue (Wu et al., 2024). This mitochondrion is packaged in an enteric gel capsule. The orally administered mitochondria are rapidly absorbed by intestinal cells and subsequently released into the bloodstream, from where they are transported to the damaged heart tissue. Modulation of the disease microenvironment by nanomitochondria not only facilitates the rapid uptake and prolonged retention of mitochondria by damaged cells, but also preserves the high functional activity of exogenous mitochondria. It would be an intriguing attempt if the chemotactic targeting ability of mitochondrial transfer to chondrocytes could be enhanced through a similar approach. Given that hyaluronic acid is frequently utilized in palliative treatment for OA through intra-articular injection, exploring its potential to deliver mitochondria presents novel approach to for OA treatment. Hydrogel is also one of the candidate carriers, and an engineered layered hydrogel with immunoreactive properties that can adapt to the bone regeneration environment and mediate targeted mitochondrial transfer between cells was recently published (Cai W. et al., 2024). This finding provides a new therapeutic strategy to promote bone regeneration and repair, and also has research value and practical applications in the treatment of OA.

Moreover, the indirect enhancement of mitochondrial transfer efficiency represents a viable strategy. The mitochondrial transfer technology based on droplet microfluidics has demonstrated efficient, high-throughput quantitative mitochondrial transfer at the single-cell level. This advancement is expected to significantly promote mitochondrial transfer in clinical applications and optimize cellular function, thereby creating new avenues for the treatment of OA (36). Currently, most of the clinical applications alter the efficiency of mitochondrial transfer by modulating key signalling pathways. For example, magnetic iron oxide nanoparticle (IONP)-activated hMSCs (Fe-hMSCs) can induce the overexpression of Cx43, thereby promoting mitochondrial transfer and enabling human placenta-derived MSCs to efficiently and safely transfer mitochondria to target cells (Huang et al., 2021). This could also be a therapeutic reference for mitochondrial transfer in OA.

### 5.4 Challenges of artificial mitochondrial transfer in the treatment of OA

Artificial mitochondrial transfer is emerging as a significant therapeutic approach in the treatment of OA, and researchers are actively investigating its potential. However, this procedure encounters several challenges. In the pre-mitochondrial transfer phase, implementers need to refine the treatment plan. OA as a syndrome has different etiologies and significant differences in the pathological stages of presentation (Glyn-Jones et al., 2015; Li et al., 2024c; Papatriantafyllou, 2024; Coryell et al., 2021). Consequently, the efficacy of artificial mitochondrial transfer in addressing specific etiologies, as well as the precise criteria for patient selection for personalized treatment, remains an unresolved issue. Moreover, the optimal therapeutic window for artificial mitochondrial transfer during disease progression to maximize efficacy remains undefined.

Moreover, although mitochondria exhibit a certain affinity for specific tissues, their targeting of chondrocytes within the human body requires enhancement (Tracy et al., 2022; Justin et al., 2024; Qinglian et al., 2003). Ensuring that mitochondria can accurately localize to damaged joint tissues or cells represents a critical issue in current research. It is reported that cells can capture purified mitochondria for aerobic respiration, which raises the possibility that non-targeted cells can capture mitochondria and affect the therapeutic effect (Borcherding et al., 2022). Additionally, artificial mitochondrial transfer may elicit an immune response because mitochondria possess mtDNA, which can be recognized by the immune system as foreign material, potentially leading to an immune attack (Tobias et al., 2019). Such a response may result in transfer failure, cause other complications, and even exacerbate the condition of OA. At present, the clinical application of mitochondrial transfer remains in its infancy, with a limited number of cases reported, thereby complicating a comprehensive assessment of its efficacy and safety (Craven et al., 2018).

Following mitochondrial transfer, it is imperative for the mitochondria to sustain stable functionality within the recipient cells. However, variations in the cellular environment of aging of the transplanted mitochondria, may lead to a gradual decline in their functionality (Yi et al., 2024). Additionally, the processes of mitochondrial fusion and degradation present significant challenges: transplanted mitochondria must successfully fuse with the recipient cells' mitochondria to fulfill their intended functions, a process that may be influenced by a variety of regulatory factors (National Academies of Sciences E and Medicine, 2016; Gammage et al., 2018). It was found that a portion of naked mitochondria can escape from recipient cells after capture, and that some donor-derived mitochondria may also be degraded by recipient cells and fail to fulfil their intended role (Cowan et al., 2017). While some studies have demonstrated positive outcomes of mitochondrial transfer in animal models, the long-term efficacy and safety of this intervention in human subjects necessitate further investigation.

## 6 Outlook

Mitochondrial dysfunction in osteoarthritis presents as a multifaceted disease mechanism, featuring diverse manifestations with interconnected regulatory networks. Future therapies targeting mitochondrial restoration represent a promising frontier for OA intervention. Given the operability and favorable biocompatibility of artificial mitochondrial transfer, this strategy appears promising as an effective intervention for future OA therapies. Artificial mitochondrial transfer can be conducted through various carriers, including stem cells, subcellular organelles, bioengineering materials, and even the direct transfer of isolated mitochondria. Additionally, targeted interventions, physical stimulation, bio-protein mediation, and modulation of signaling pathway can also be employed to enhance the efficiency of mitochondrial transfer.

Currently, the research directions pertaining to artificial mitochondrial transfer are varied, offering numerous opportunities for achieving the objectives of mitochondrial transfer. Nevertheless, investigations in this domain remain in the nascent stages, necessitating a clarification of the differences in responses observed between animal models and humans subjects. It is imperative to validate these findings through more experimental data prior to advancing to clinical applications. Advancing phase-targeted osteoarthritis therapies while resolving key technical challenges, including mitochondrial delivery optimization and therapeutic durability, represents critical research priorities. With continued investigation, artificial mitochondrial transfer is anticipated to provide new therapeutic strategies for OA, accommodating its diverse stages and underlying causes.

## Glossary

AdMSC-Exos: Exosomes from adipose-derived mesenchymal stem cells
ADP: Adenosine diphosphate
AKT: Protein kinase B
AMP: Adenosine monophosphate
AMPK: Adenosine 5′-monophosphate (AMP)-activated protein kinase
ATP: Adenosine triphosphate
BMSCs: Bone marrow derived mesenchymal stem cells
CaMKII: Calcium/calmodulin-dependent protein kinase II
Cx43: Connexin 43
DAMP: Danger-associated molecular pattern
DDS: Drug delivery system
DMT: Disease-modifying therapy
DRP1: Dynamin-related protein one
ECM: Extracellular matrix
ERK: Extracellular regulated protein kinases
ETC: Electron transport chain
EV: Extracellular vesicle
Fe-hMSCs: Magnetic iron oxide nanoparticle (IONP)-activated human MSCs
Fe/S clusters: Iron-Sulfur clusters
FIS1: Fission protein 1
FMCs: Fusogenic mitochondrial capsules
GABPA: Guanine-adenine(GA)binding protein transcription factor
GJA1-20k: Gap junction protein alpha 1–20 kDa
GJC: Gap junction channels
GMP-AMP: Guanosine monophosphate-adenosine monophosphate
GTPase: Guanosine triphosphate
H3K27me3: Histone H3 lysine 27 trimethylation
HSCs: Haematopoietic stem cells
IL-1β: Interleukin-1 beta
M1 Macrophages: Classically activated macrophages
M2 Macrophages: Alternatively activated macrophages
MAPK: Mitogen-activated protein kinase
MDVs: Mitochondrial-derived vesicles
MIA: Monoiodoacetate
Miro 1: Mitochondrial Ras homology guanosine triphosphate one
MMPs: Matrix metalloproteinases
mPTP: Mitochondrial permeability transition pore
MS: Multiple sclerosis
MSCs: Mesenchymal stem cells
MT: Mitochondria
mtDNA: Mitochondrial deoxyribonucleic acid
mTOR: Mammalian target of rapamycin
NAD+: Nicotinamide adenine dinucleotide (oxidized form)
NADH: Nicotinamide adenine dinucleotide (reduced form)
NLRP3: Nucleotide-binding domain(NOD)-like receptor family pyrin domain containing 3
OA: Osteoarthritis
OAI: Osteoarthritis initiative
OXPHOS: Oxidative phosphorylation
PD: Parkinson’s disease
PGC-1α: Peroxisome proliferator-activated receptor-γ coactivator-1α
PGC-1β: Peroxisome proliferator-activated receptor gamma co-activator 1β
Phf8: Plant homeodomain finger protein 8
PI3K: Phosphatidylinositol 3 kinase
PINK1: Phosphatase and tensin homolog induced putative kinase one
PN-101 Mitochondria: Human umbilical cord mesenchymal stem cell-derived mitochondria
PPARγ: Peroxisome proliferator-activated receptor γ
PRP: Platelet-rich plasma
ROS: Reactive oxygen species
SE: Status epilepticus
siRNA: Small interfering ribonucleic acid
SIRT: Sirtuins
SLSMD: Single large-scale mitochondrial deoxyribonucleic acid deletion
SVF: Stromal vascular fraction
T-cells: Thymus dependent lymphocyte
TFAM: Nuclear-encoded mitochondrial transcription factor A
TNF-α: Tumor necrosis factor-alpha
TNTs: Tunneling nanotubes
UC: Umbilical cord
YY-1: Yin yang one
ρ0 cells: Cells depleted of mitochondrial deoxyribonucleic acid
